# TNFα Mediates Inflammation-Induced Effects on *PPARG* Splicing in Adipose Tissue and Mesenchymal Precursor Cells

**DOI:** 10.3390/cells11010042

**Published:** 2021-12-24

**Authors:** Simona Cataldi, Marianna Aprile, Daniela Melillo, Inès Mucel, Sophie Giorgetti-Peraldi, Mireille Cormont, Paola Italiani, Matthias Blüher, Jean-François Tanti, Alfredo Ciccodicola, Valerio Costa

**Affiliations:** 1 Institute of Genetics and Biophysics ‘‘Adriano Buzzati-Traverso’’, CNR, Via P. Castellino 111, 80131 Naples, Italy; simona.cataldi@igb.cnr.it (S.C.); marianna.aprile@igb.cnr.it (M.A.); alfredo.ciccodicola@igb.cnr.it (A.C.); 2 Institute of Biochemistry and Cell Biology, CNR, Via P. Castellino 111, 80131 Naples, Italy; daniela.melillo@ibbc.cnr.it (D.M.); paola.italiani@ibbc.cnr.it (P.I.); 3 Université Côte d’Azur, Inserm UMR1065, C3M, Team Cellular and Molecular Pathophysiology of Obesity, 06204 Nice, France; ines.mucel@etu.univ-cotedazur.fr (I.M.); sophie.giorgetti-peraldi@inserm.fr (S.G.-P.); cormont@unice.fr (M.C.); tanti@unice.fr (J.-F.T.); 4 Medical Department III-Endocrinology, Nephrology and Rheumatology, University of Leipzig, 04103 Leipzig, Germany; matthias.blueher@medizin.uni-leipzig.de; 5 Helmholtz Institute for Metabolic, Obesity and Vascular Research (HI-MAG) of the Helmholtz Zentrum München at the University of Leipzig and University Hospital Leipzig, Philipp-Rosenthal-Str. 27, 04103 Leipzig, Germany; 6 Department of Science and Technology, University of Naples ‘‘Parthenope’’, 80143 Naples, Italy

**Keywords:** *PPARG* splicing, dominant negative isoform, inflammation, TNFα, adipocyte precursors, hypertrophic obesity

## Abstract

Low-grade chronic inflammation and reduced differentiation capacity are hallmarks of hypertrophic adipose tissue (AT) and key contributors of insulin resistance. We identified PPARGΔ5 as a dominant-negative splicing isoform overexpressed in the AT of obese/diabetic patients able to impair adipocyte differentiation and PPARγ activity in hypertrophic adipocytes. Herein, we investigate the impact of macrophage-secreted pro-inflammatory factors on *PPARG* splicing, focusing on PPARGΔ5. We report that the epididymal AT of LPS-treated mice displays increased PpargΔ5/cPparg *ratio* and reduced expression of *Pparg*-regulated genes. Interestingly, pro-inflammatory factors secreted from murine and human pro-inflammatory macrophages enhance the PPARGΔ5/cPPARG *ratio* in exposed adipogenic precursors. TNFα is identified herein as factor able to alter *PPARG* splicing—increasing PPARGΔ5/cPPARG *ratio*—through PI3K/Akt signaling and SRp40 splicing factor. In line with in vitro data, TNFA expression is higher in the SAT of obese (vs. lean) patients and positively correlates with PPARGΔ5 levels. In conclusion, our results indicate that inflammatory factors secreted by metabolically-activated macrophages are potent *stimuli* that modulate the expression and splicing of *PPARG*. The resulting imbalance between canonical and dominant negative isoforms may crucially contribute to impair PPARγ activity in hypertrophic AT, exacerbating the defective adipogenic capacity of precursor cells.

## 1. Introduction

Obesity is a chronic, relapsing progressive disease considered a driving force for insulin resistance and type-2 diabetes (T2D) [1,2]. Low-grade chronic inflammation in adipose tissue (AT) crucially contributes to obesity-related metabolic dysfunctions [3,4,5]. Indeed, the energy *surplus* can induce the enlargement of adipocyte size (hypertrophy), in turn leading to hypoxia, mechanical stress of the extracellular matrix, cell death and the secretion of pro-inflammatory cytokines [6,7,8,9]. These events trigger a chronic inflammatory response, stimulating the infiltration of macrophages (MΦ), T and dendritic cells [3,4,5,9,10]. In turn, the substantial accumulation and polarization of MΦ into pro-inflammatory and metabolically activated cells—associated with qualitative and quantitative genetic and epigenetic remodeling—increases local and systemic levels of cytokines and chemokines, correlated with insulin resistance and T2D (e.g., TNFα, IL-6, IL1β, CCL4) [11,12,13,14,15]. Indeed, MΦ-secreted factors impair the insulin-responsiveness of AT-residing adipocytes [16,17] and the differentiation capacity of mesenchymal adipogenic precursors [12,18]. These molecules reduce the expression and activity of the peroxisome proliferator-activated receptor γ (PPARγ) [19,20,21,22], the master regulator of adipogenesis and metabolic homeostasis [23,24,25,26,27,28]. However, the exact molecular mechanisms determining the functional impairment of PPARγ in insulin-resistant AT have not been fully elucidated. Our recent identification and characterization of PPARγΔ5, a naturally occurring dominant negative isoform of PPARγ generated by exon 5 skipping, revealed a new layer in the regulation of *PPARG* [29]. Indeed, increased levels of PPARγΔ5—measured in the subcutaneous AT (SAT) of patients with overweight and obesity—impairs the in vitro differentiation ability of adipocyte precursor cells [29]. Increased PPARGΔ5/cPPARG *ratio* was also measured in our recently generated in vitro model of human hypertrophic-like adipocytes [30]. Moreover, the PPARGΔ5/cPPARG relative amount in the SAT of overweight and obese patients correlated positively with BMI [29] and negatively with *SLC2A4* (i.e., GLUT4) mRNA levels [30], suggesting a contribution of PPARγΔ5 in hypertrophic obesity. 

Hence, in this work, we investigated whether the balance between canonical and dominant negative PPARγ isoforms is affected by pro-inflammatory factors and if such alteration contributes to the impaired neo-adipogenesis and insulin-resistance in hypertrophic AT. Herein, we demonstrate that MΦ-secreted pro-inflammatory factors affect *PPARG* splicing, increasing the PPARGΔ5/cPPARG *ratio*. Notably, we report that TNFα induces the activation of the serine/arginine-rich protein 40 (SRp40) and PI3K/Akt signaling, representing the most powerful inflammatory *stimulus* able to modulate *PPARG* expression and splicing. Moreover, *TNFA* and PPARGΔ5 mRNA levels show a positive correlation in the SAT of obese patients, further supporting *PPARG* splicing alteration as a feature of inflamed AT. Our work reveals a previously unrecognized role of macrophage-secreted factors on *PPARG* regulation, indicating the inflammation-mediated alteration of *PPARG* splicing in adipogenic precursors as a new potential contributor to the defective neo-adipogenesis of hypertrophic adipose tissue.

## 2. Materials and Methods

### 2.1. Human Samples 

Adipose tissue samples were obtained from donors to the Leipzig Obesity Bio Bank (n = 55; mean age = 57.6 ± 16.7 years; mean BMI = 32.3 ± 9.9 kg/m^2^) undergoing bariatric surgery [31,32] and available through previously reported collaborations [29,30]. The study has been reviewed and approved by the ethics committee of Leipzig University, Germany (approval numbers: 159-12-21052012 and 017-12-23012012), and carried out in accordance with the Declaration of Helsinki, the Bioethics Convention (Oviedo) and the EU Directive on Clinical Trials (Directive 2001/20/EC). Patients were randomly selected from the human tissue biopsy bank held by Prof. Bluher, with more than 3700 subjects having a wide range of overweight and obesity (BMI range: 13–129 kg/m^2^), body fat distribution, insulin sensitivity and glucose tolerance. The random selection of samples, as well as the exclusion criteria of individuals were applied as described in Aprile et al. (2018). Briefly, individuals with the following criteria were excluded: age <18 years, obesity secondary to endocrine diseases, gastrointestinal inflammatory diseases, risk of upper gastrointestinal bleeding, mental disorders, previous or current malignancies, habitual consumption of alcohol or drugs. Patients were categorized according to their BMI into three subgroups: lean (BMI ≤ 25 kg/m^2^; n = 14), overweight (25 < BMI < 30 kg/m^2^; n = 17) and obesity (BMI ≥ 30 kg/m^2^; n = 24).

### 2.2. Animals

Epididymal AT samples were isolated from C57BL/6J mice *ip*-injected with LPS (2 µg/g of body weight, InvivoGen, San Diego, USA) or with vehicle (NaCL 0.9%) for 5 h. Mice and adipose tissue samples were already described in a previous publication [33]. The study was conducted according to the Principles of Laboratory Animal Care (NIH publication no. 85–23, revised 1985) and the European Union guidelines on animal laboratory care. All procedures were approved by Animal Care Committee of the Faculty of Medicine of Nice-Sophia Antipolis University, Nice, France, and the French ministry of national education (#05116.02 and #201505&9143792_v2).

### 2.3. Cell Cultures

Human in vitro monocytes, i.e., THP-1 (Cat#TIB-202), h*TERT* immortalized adipose-derived mesenchymal stem cells (hMSCs; Cat# SCRC4000)were purchased from the American Type Culture Collection (ATCC). HMSCs were cultured in DMEM-F12 (1:1) supplemented with 10% American fetal bovine serum (FBS), 2 mM glutamine, 30 units/mL penicillin and 30 µg/mL streptomycin. THP-1 cells were grown in suspension with RPMI supplemented with 10% American fetal bovine serum (FBS), 2 mmol/L glutamine, 100 units/mL penicillin and 100 units/mL streptomycin. 

Human monocytes were isolated from the fresh buffy coats of healthy donors recruited at the Transfusional Center of the Hospital Policlinico at “Federico II” University of Naples (Italy) after informed consent according to the Declaration of Helsinki. PBMCs were obtained by density centrifugation over Ficoll-Paque plus (GE Healthcare, Bio-sciences AB, Uppsala, Sweden), and, subsequently, CD14+ monocytes were isolated from PBMCs using anti-CD14 antibody-bearing magnetic microbeads (Miltenyi Biotec, Bergisch-Gladbach, Germany), according to the manufacturer’s instructions. Primary monocytes were resuspended in RPMI-1640 medium (GIBCO, Life Technologies, Carlsband, CA, USA) containing 5% heat-inactivated human AB serum (Lonza, Pearland, TX, USA) and 50 mg/mL gentamicin (Sigma-Aldrich Inc., St. Louis, MO, USA) and cultured at a density of 0.5 × 10^6^ cells/mL/well in 24-well culture plates (Costar, Corning, NY, USA). The purity of isolated cells was determined microscopically after cytocentrifugation and differential staining with a modified Wright-Giemsa dye (Diff Quik, Medion Diagnostics AG, Düdingen, Switzerland), while viability was determined by trypan blue dye exclusion. Only preparations with >98% purity was used.

The J774A.1 mouse macrophage cell line (Cat#85011428) was obtained from the European Collection of Authenticated Cell Culture and 3T3-L1 mouse fibroblasts (Cat#CL-173^TM^) from ATCC. J774A.1 cells were cultured in DMEM supplemented with 5% American fetal bovine serum (FBS), 2 mmol/L glutamine, 100 units/mL penicillin and 100 units/mL streptomycin. 3T3-L1 fibroblasts were cultured in DMEM supplemented with 10% of newborn calf serum, 2 mmol/L glutamine, 100 units/mL penicillin and 100 units/mL streptomycin. 

All above-mentioned cell cultures, except for 3T3-L1 cells, were maintained in a humidified atmosphere of 95% air and 5% CO_2_ at 37 °C. 3T3-L1 cells were maintained in a humidified atmosphere of 95% air and 7% CO_2_ at 37 °C.

All media, sera and antibiotics were purchased from Thermo Fisher Scientific (Waltham, MA, USA).

### 2.4. Human Adipocyte Differentiation 

HMSCs were differentiated in mature adipocytes as previously described [29,30]. Briefly, cells were plated at 3 to 4 × 10^3^/cm^2^ density and grown to maximum confluence (i.e., timepoint, T = 0 h). Adipocyte differentiation was induced by alternatively using two different mixes: an adipocyte differentiation induction mix and a maintaining mix (AIM and AMM, respectively). AIM contains insulin 850 nM (Humulin R, Lilly, Indianapolis, IN, USA, Cat#AIC025707011/M), dexamethasone 10 µM (Sigma-Aldrich Inc., St. Louis, MO, USA, Cat#D4902), 3-isobutyl-1-methylXanthine 0.5 µM (Sigma-Aldrich Inc., St. Louis, MO, USA, Cat#I7018), biotin 33 µM (Sigma-Aldrich Inc., St. Louis, MO, USA, Cat#B4639), pantothenate 17 µM (Sigma-Aldrich Inc., St. Louis, MO, USA, Cat#P5155) and rosiglitazone 1 µM (Sigma-Aldrich Inc., St. Louis, MO, USA, Cat#R2408). AMM consists of 850 nM insulin and 1 µM rosiglitazone. Cells reached complete adipocyte differentiation within 19–21 days after the first induction (T = 21 d).

### 2.5. Differentiation of Primary and InVitro Human Macrophages

THP-1 human monocytes were seeded in a complete culture medium supplemented with 50ng/mL of Phorbol 12-Myristate 13-Acetate (PMA, Sigma-Aldrich Inc, St. Louis, MO, USA, Cat#P8139) at 10 × 10^6^/10 mL density in a 10 cm plate. The medium, containing 50 ng/mL of PMA, was replaced every two days until a complete macrophage differentiation was reached (i.e., 6 days after induction). 

In order to generate in vitro monocyte-derived macrophages, fresh primary monocytes—isolated as described above—were cultured for 7 days in complete medium supplemented with 50 ng/mL M-CSF (R&D Systems, Minneapolis, MN) and replaced at day 3. Terminally differentiated macrophages (i.e., 8 days after differentiation induction) were incubated for 24 h in fresh complete medium (i.e., control cells) or supplemented with LPS (10 ng/mL) of *Escherichia coli* O55:B5 (Sigma-Aldrich Inc., St. Louis, MO, USA) and IFNγ 20 ng/mL (R&D Systems, Minneapolis, MN, USA) for cell activation in pro-inflammatory macrophages or supplemented with IL-10 (20 ng/mL; R&D Systems, Minneapolis, MN, USA) to obtain anti-inflammatory macrophages.

### 2.6. Conditioned Medium Preparation

The conditioned medium of human macrophages differentiated from THP-1 was obtained stimulating—or not—the cells with LPS (20 ng/mL; Sigma-Aldrich Inc, St. Louis, MO, USA, Cat# L2630) in RPMI supplemented with 0.5% bovine serum albumin. After 24 h, the medium was collected and centrifuged at 1000× *g* rpm for 10 min, and cell-free supernatant was used for treating hMSCs, as described below. Similarly, the conditioned media of primary macrophages—both pro-inflammatory (i.e., LPS/IFNγ-induced macrophages) and anti-inflammatory (IL10-induced macrophages)—were obtained by collecting supernatants after 24 h of stimulation (as described above) and centrifugation at 1000× *g* rpm for 10 min at 4 °C. Finally, J774A.1 mouse macrophages were stimulated or not with 0.5 ng/mL of LPS in DMEM supplemented with 5% FBS for 24 h, and the medium was collected and centrifuged at 1300× *g* rpm for 5 min. 

### 2.7. Murine Adipocyte Differentiation 

3T3-L1 mouse fibroblasts were cultured at confluence and induced to differentiate in mature adipocytes by two differentiation mixes. Thus, control (i.e., DMEM-5% FBS or DMEM-5% FBS plus LPS 0.5 ng/mL) or conditioned media (collected from LPS stimulating and not J774A.1 macrophages) were supplemented by isobutyl methylxanthine 0.5 µM (Sigma-Aldrich Inc., St. Louis, MO, USA, Cat# I5879), dexamethasone 0.25 µM (Sigma-Aldrich Inc., St. Louis, MO, USA, Cat# D1756), rosiglitazone 10 µM (Enzo Life Science, Farmingdale, NY, USA, Cat# ALX-350-125) and insulin 5 µg/mL (Humulin, Lilly, Indianapolis, IN, USA, Cat# HI0290). After two days, the cells were treated with insulin (5 µg/mL) and rosiglitazone (10 µM) for an additional two days. Then, fresh medium—i.e., without the adipogenic cocktail—was added until the adipocyte phenotype appeared in more than 90% of the cells (i.e., 8 days after induction) by microscopic visualization.

### 2.8. Oil Red O Staining

Lipid accumulation was measured by Oil red O staining, as previously described [20,29]. In detail, murine adipocytes (i.e., 8 days after adipogenesis induction) were fixed with 4% paraformaldehyde for 20 min, washed three times with PBS and then stained with Oil red O (Sigma-Aldrich Inc., St. Louis, MO, USA, Cat# O0625) for 15 min. Cells were washed, and Oil red O was eluted by isopropanol 100%. The quantification of accumulated lipids was performed by optical density measurement at 510 nm using the spectrophotometer. The background signal was estimated in undifferentiated cells and subtracted from optical density values. The significance of the differences between the samples was calculated by a two-tailed one sample Student’s *t* test.

### 2.9. Cell Treatments

HMSCs (i.e., T = 0 h) or hMSCs-derived mature adipocytes (i.e., T = 21 d after adipocyte-differentiation induction) were treated for 24 h with conditioned medium collected by human macrophages (differentiated from THP-1 as above described) or with RPMI 0.5% BSA supplemented or not with 20 ng/mL LPS, used as control. Moreover, hMSCs (i.e., T = 0 h) were treated for 24 h with culture supernatants of primary not-polarized macrophages or were activated by LPS/IFNγ or IL-10. 

To investigate *PPARG* mRNA half-life, hMSCs were treated for 3 h with 2 or 5 mg/mL of actinomycin D (Sigma-Aldrich Inc., St. Louis, MO, USA, Cat# A1410) in complete culture medium. Cells treated with the vehicle (i.e., dimethyl sulfoxide, DMSO) were used as control. 

HMSCs were treated with human recombinant cytokines or with the vehicle (i.e., PBS) in DMEM-F12 supplemented with 1% FBS. Specifically, cells were treated for 24 h (for gene expression analysis) or for 30 min (for protein assays) with 10 ng/mL of IL-6 (Thermo Fisher Scientific, Waltham, MA, USA, Cat# PHC0066), IL-8 (Preprotech, Rocky Hill, NJ, USA, Cat#200-08), IL-1β (Preprotech, Rocky Hill, NJ, USA, Cat# 200-01B) cytokines or increasing doses of TNFα protein (i.e., 5, 10 or 20 ng/mL; SinoBiological, Beijing, China, Cat# 10602-HNAE-50).

Conditioned medium collected by THP-1-derived macrophages was incubated with 0.5 mg/mL of human neutralizing antibody against TNFα (R&D system, Minneapolis, USA, Cat# MAB610-SP) or anti-IgG1 Isotype (R&D system, Minneapolis, MN, USA, Cat# MAB002) for 2 h at 4 °C. Then, the medium supplemented with antibodies was added to hMSCs for 24 h for mRNA expression analysis and 30 min for protein assays. Cells treated with RPMI 0.5% BSA plus anti-IgG1 Isotype were used as control.

Moreover, hMSCs were treated for 2 h with DMEM 1% FBS supplemented with 300nM of Wortmannin (Thermo Fisher Scientific, Waltham, Massachusetts, USA, Cat# PHZ1301), KHCB19 (Santa Cruz Biotechnology, Dallas, TX, USA, Cat# 1354037-26-5) or the vehicle (i.e., DMSO). Then, cells were exposed to fresh medium supplemented with 10 nM of Wortmannin/KHCB19/vehicle and human recombinant TNFα protein (10 or 20 ng/mL) for an additional 30 min to analyze protein phosphorylation or 24 h for mRNA expression analysis. 

In addition, hMSCs were treated for 24 h with conditioned medium collected from THP-1-derived macrophages plus SRPIN340 10 µM (StressMarq Biosciences Inc., Victoria, Canada, SIH-514, Cat# 218156-96-8) or with the vehicle (i.e., DMSO). 

3T3-L1 mouse fibroblasts (i.e., T = 0 h) were exposed for 24 h to DMEM-1% FBS supplemented with 10 µg/mL of mouse recombinant TNFα (Preprotech, Rocky Hill, NJ, USA, Cat# 315-01A), IL-1β (Preprotech, Rocky Hill, NJ, USA, Cat# 211-11B) cytokines or with the vehicle (i.e., PBS).

For all treatments, cells were starved for 18 h with culture medium without serum.

### 2.10. Cell Transfection

HMSCs were transfected with two different siRNAs designed against *SRSF5* (IDT, Coralville, Johnson County, IA, USA, hs.Ri.SRSF5.13.1) or with scrambled siRNAs, by using Lipofectamine 3000 (Thermo Fisher Scientific, Waltham, Massachusetts, MN, USA, Cat# L3000001), according to the manufacturer’s instructions. Cell transfection was carried out in culture medium without antibiotics and serum. After 6 h, transfected cells were fed with medium supplemented with 10% FBS. The efficiency of silencing was calculated by quantitative PCR assays (qPCR) after 24 and 48 h of cell transfection, using different siRNAs in single or in combination. The siRNA hs.Ri.SRSF5.13.1 (40 nM) induced the silencing of *SRSF5* with estimated efficiency of about 75% and was selected for further analysis. After 18 h of transfection, *SRSF5*-knockdown cells were treated for 24 h with human recombinant TNFα cytokine (10 or 20 ng/mL) or with the conditioned medium of THP-1-derived macrophages. 

### 2.11. RNA Isolation, RT-PCR and Quantitative PCR

Epididymal adipose tissue from C57BL/6J mice was homogenized by using Precellys tissue homogenizer. Total RNA was isolated from homogenized tissues and cell lines byTRIzol Reagent (Thermo Fisher Scientific, Waltham, Massachusetts, MN, USA, Cat# 15596026), according to the manufacturer’s instructions. Isolated RNA was quantified with NanoDrop spectrophotometer and was reverse transcribed using “High-Capacity cDNA Reverse Transcription kit” (Thermo Fisher Scientific, Waltham, Massachusetts, MN, USA, Cat# 4368813). Gene expression analysis was performed by quantitative PCR assays using iTaq Universal Sybr Green Supermix (Bio-Rad, Hercules, CA, USA, Cat# 1725125), according to the manufacturer’s instructions, on a CFX Connect Detection System (Bio-Rad, Hercules, CA, USA). The specific primer pairs were designed using the Oligo 4.0 program and are listed in Table S1. The specificity of the amplification reaction was confirmed by melt curve analysis. *PPIA*, *RPS23* and *36b4* were selected as reference genes for analyzing human and mouse samples, respectively. Relative expression analysis was performed using the 2^−^^ΔΔCt^ method, except for the analysis of *TNFA* expression levels assessedin the Leipzig cohort by the ΔCt method. All reactions were performed in duplicate in at least three independent experiments.

### 2.12. Western Blot

Lysates were harvested from hMSCs by a RIPA lysis buffer containing Halt Protease and Phosphatase Inhibitor Cocktail (Thermo Fisher Scientific, Waltham, Massachusetts, USA). Protein content was determined using the Bradford protein assay (BioRad, Hercules, CA, USA, Cat# 5000205), and 60 µg of lysates were separated on 12% SDS-PAGE and transferred to PDVF membranes. Membranes were blocked with Tris-buffered saline–Tween (TBST) containing 4% non-fat dried milk and incubated overnight with primary antibodies against the phospho-epitope of SR proteins (1:500, Merck Millipore, Darmstadt, Germany, clone 1H4, Cat# MABE50), the phospho-Akt at Ser473 residue (1:800; Biolegend, San Diego, CA, USA, Cat# 649001) or total Akt1 (1:1000; Biolegend, San Diego, CA, USA, Cat# 680302). After incubation for 1.5 h at room temperature with anti-IgG (HRP)-conjugated secondary antibody (1:4000, Bio-Rad, Hercules, CA, USA, Cat# 170-6515/A28175), signal detection was performed by Pierce ECL Western Blotting Substrate (Thermo Fisher Scientific, Waltham, MA, USA). The shown autoradiographs are representative of at least three independent experiments. Densitometric data analysis was performed on pixel density by GelQuant.NET software (www.biochemlabsolutions.com (accessed on 31 January 2021)). Intensity values were normalized using Hsp90 as the housekeeping gene and compared to signals detected in reference samples. 

### 2.13. ELISA

Levels of the inflammatory cytokines were determined by ELISA (R&D Systems, MN, USA) in cell-free supernatants according to the manufacturer’s instructions. The absorbance of assay wavelength was measured at 450 nm using a Cytation 3 imaging reader (BioTek, Winooski, VT, USA).

### 2.14. Statistics

All data are expressed as means ± SEM, except for expression data in human biopsies that are expressed as median ± DEVST. All assays were performed at least in triplicate. A Shapiro–Wilk test (“shapiro.test function”) implemented in R language was used for assessing the normal distribution of data. Statistical significance of differences between testing and control samples was evaluated by a two-tailed (one or two sample) Student’s *t* test (GraphPad Software Inc., La Jolla, CA, USA). Gene expression differences in the German cohort were analyzed by a Mann–Whitney U test. A linear model implemented in R language (lmp function) was used for correlation analysis, as described elsewhere [29]. Boxplots showing *TNFA* mRNA expression analysis were generated in R using the ggplot2 library and custom scripts. Differences between testing and control samples were defined as significant as *p* value ≤ 0.05.

## 3. Results

### 3.1. The Inflammatory Milieu Affects Pparg Expression and Splicing In Vitro and In Vivo

Pro-inflammatory *stimuli* suppress PPARγ transcriptional activity in murine primary adipocytes and pre-adipocyte cell lines linking the inflammation of AT to insulin resistance in obese patients [19]. We recently reported the increase of PPARGΔ5 and of the PPARGΔ5/cPPARG ratio in the SAT of obese patients [29], and a tight association with hypertrophic—rather than “metabolically-healthy” hyperplastic—obesity and its related metabolic alterations [30].

**Figure 1:**
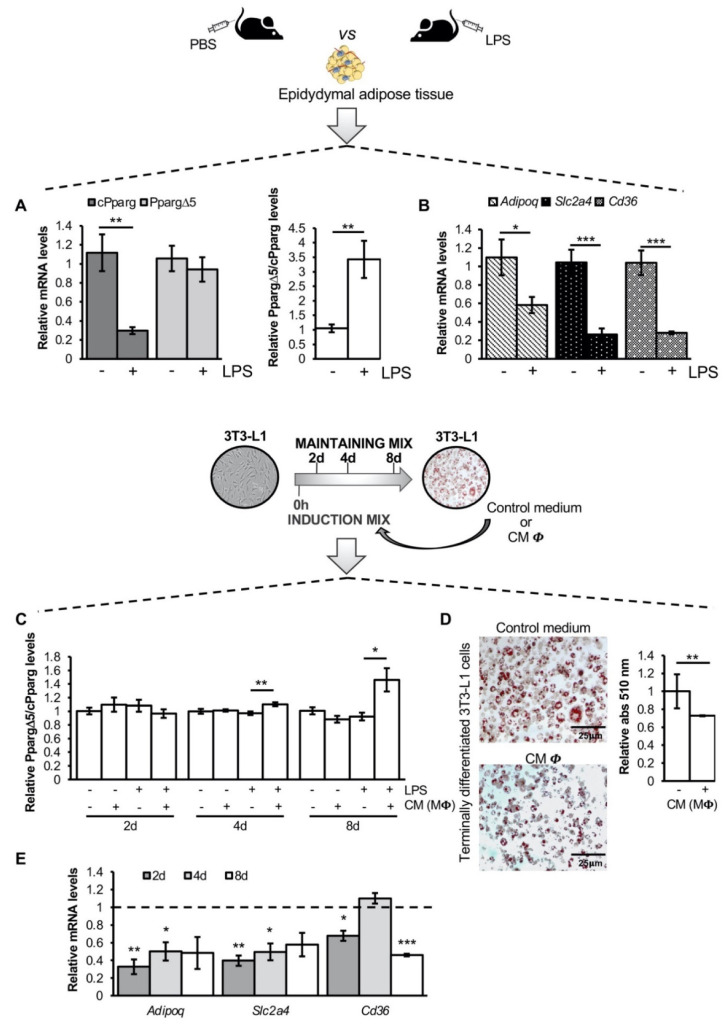
Inflammation affects *Pparg* expression and splicing in vitro and in vivo: (**A**) Relative mRNA quantification (qPCR) of canonical Pparg transcripts (cPparg), PpargΔ5 (left panel), PpargΔ5/cPparg ratio (right panel) and Pparγ target genes (i.e., *Adipoq*, *Slc2a4* and *Cd36*, (**B**)) in epididymal adipose tissue of C57BL/6JB-LPS injected mice (n = 6). Epididymal adipose tissue from control mice (n = 7) was used as reference samples and *36b4* as reference gene. Data are reported as mean ± SEM of independent experiments. * *p* ≤0.05, ** *p* ≤ 0.01 and *** *p* ≤ 0.001. (**C**) Relative mRNA quantification (qPCR) of the PpargΔ5/cPparg ratio at different time points of 3T3-L1 adipocyte differentiation (i.e., 2, 4 and 8 days upon differentiation induction) carried out in presence of conditioned medium (CM) of J774.A1 macrophages (MΦ) activated and not with LPS. 3T3-L1 differentiated in mature adipocytes with control medium with or without LPS were used as reference samples. *36b4* was used as reference gene. Data are reported as mean ± SEM of at least six independent experiments. * *p* ≤ 0.05, ** *p* ≤ 0.01. (**D**) Representative images of 3T3-L1 differentiated in mature adipocytes (i.e., 8 days upon differentiation induction) in presence and not of CM collected from LPS activated J774.A1 MΦ (scale bar 25 µm). Lipid accumulation was measured by optical determination of Oil red O staining and reported in the bar graph. 3T3-L1 terminally differentiated in mature adipocytes with control medium plus LPS were used as reference samples. Data are reported as mean ± SEM of three independent experiments. ** *p* ≤ 0.01. (**E**) Relative mRNA quantification (qPCR) of Pparγ target genes (i.e., *Adipoq*, *Slc2a4* and *Cd36*) at different time points of 3T3-L1 adipocyte differentiation (i.e., 2, 4 and 8 days upon differentiation induction) carried out in presence of CM of LPS activated J774.A1 cells. 3T3-L1 differentiated in mature adipocytes with control medium plus LPS were used as reference samples (dotted line) and *36b4* as reference gene. Data are reported as mean ± SEM of at least three independent experiments. * *p* ≤ 0.05, ** *p* ≤ 0.01 and *** *p* ≤ 0.001.

To assess in vivo whether inflammation—hallmark of hypertrophic obesity—affects *PPARG* splicing, we measured the relative abundance of canonical (cPparg) and dominant negative (PpargΔ5) Pparg transcripts in the epididymal AT of a well-established mouse model of systemic inflammation (previously described in Pastor et al., 2017) [33]. As expected, *Pparg* transcription is markedly repressed (about 70%; *p* = 0.0027) in the epididymal fat of LPS-injected lean mice (Figure 1A, left panel) in line with previous findings [19]. Conversely, PpargΔ5 mRNA levels are not affected (Figure 1A, left panel), determining in turn a 3.5-fold increase (*p* = 0.024) of PpargΔ5/cPparg ratio (Figure 1A, right panel). This finding points out a previously unrecognized effect of inflammation on *Pparg* splicing in the AT. Accordingly, in line with the downregulation of cPparg levels and increased PpargΔ5/cPparg ratio, the expression of *Adipoq*, *Slc2a4* and *Cd36*—validated Pparγ target genes—is significantly reduced (Figure 1B), as we similarly observed in human lipid-engulfed hypertrophic adipocytes [30]. 

Moreover, our previous findings indicate that high PPARγΔ5 levels are associated with impaired adipogenic capacity of precursor cells [29], which represents a hallmark of hypertrophic obesity. Therefore, we explored *Pparg* splicing in murine precursor cells differentiating toward mature adipocytes in presence of a pro-inflammatory microenvironment. Hence, 3T3-L1 cells were induced to differentiate in the presence of conditioned media (CM) collected from murine J774.A1 (macrophage-like, MΦ cell line) activated and not with LPS. Interestingly, the analysis of the relative variation of all *Pparg* transcripts along the adipogenic process reveals that only the secretome of LPS-activated MΦ markedly affects *Pparg* splicing, inducing an increase of PpargΔ5/cPparg ratio in terminally differentiated adipocytes (1.5-fold, *p* = 0.03; Figure 1C and Figure S1A,B). As a consequence, pro-inflammatory microenvironment induces an impairment of the adipogenic differentiation (Figure 1D) paralleled by the downregulation of Pparγ target genes (Figure 1E), according to in vivo data (Figure 1B).

These results indicate that the inflammatory microenvironment of the AT affects *Pparg* splicing, altering the relative amount between canonical and dominant negative isoforms.

### 3.2. Pro-Inflammatory Macrophage Secretome Perturbs PPARG Splicing in Human Mesenchymal Stem Cells

**Figure 2:**
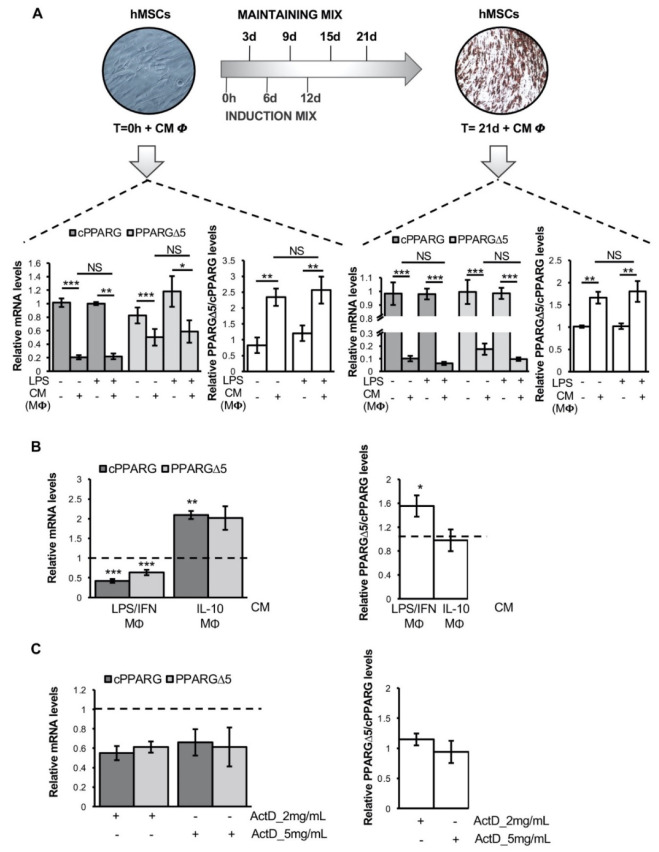
Human pro-inflammatory macrophages secretome perturbs *PPARG* splicing in human mesenchymal stem cells: (**A**) Representative images (upper panel) of human mesenchymal stem cells (hMSCs) at an undifferentiated stage (i.e., 0 h) and completely differentiated in mature adipocytes (i.e., 21 days upon differentiation induction) by using an induction and a maintaining mixes as reported on the timeline. hMSCs at 0 h and 21 d after adipocyte differentiation were treated for 24 h with conditioned medium (CM) of THP-1 macrophages (MΦ) activated and not with LPS. Below are reported relative mRNA levels (qPCR) of cPPARG, PPARGΔ5 and the PPARGΔ5/cPPARG ratio in hMSCs at 0 h (left panel) and in hMSCs at 21 d (right panel) treated with CM of MΦ. hMSCs at 0 h or at 21 d treated with control medium supplemented or not with LPS were used as reference samples and *PPIA* as reference gene. Data are reported as mean ± SEM of at least three independent experiments. * *p* ≤ 0.05, ** *p* ≤0.01 and *** *p* ≤ 0.001. (**B**) Relative mRNA quantification (qPCR) of cPPARG, PPARGΔ5 (left panel) and the PPARGΔ5/cPPARG ratio (right panel) in hMSCs at the undifferentiated stage treated for 24 h with the CM of primary monocyte-derived macrophages polarized in the pro-inflammatory (i.e., LPS/IFNγ-inducedMΦ) or anti-inflammatory (IL10-induced MΦ) type. hMSCs at 0 h treated with the CM of non-polarized primary monocyte-derived macrophages were used as a reference sample (dotted lines) and *PPIA* as a reference gene. Data are reported as mean ± SEM of at least three independent experiments. * *p* ≤ 0.05, ** *p* ≤ 0.01 and *** *p* ≤ 0.001. (**C**) Relative mRNA quantification (qPCR) of cPPARG, PPARGΔ5 (left panel) and the PPARGΔ5/cPPARG ratio (right panel) in hMSCs at 0 h treated for 3 h with 2 or 5 mg/mL of actinomycin D. hMSCs at 0h treated with vehicle (i.e., DMSO) were used as a reference sample (dotted lines). *PPIA* was used as reference gene. Data are reported as mean ± SEM of at least three independent experiments.

To determine whether inflammation also affects the *PPARG* splicing pattern in humans—similarly to that observed in mouse models—the relative abundance of canonical and dominant negative *PPARG* transcripts was measured in hMSCs and in vitro-differentiated adipocytes exposed to the conditioned media of human MΦ differentiated from THP-1 monocytes activated and not with LPS (Figure 2A, upper panel). As shown in Figure 2A (lower left panel), the exposure of undifferentiated hMSCs to conditioned media of LPS-activated MΦ (THP-1) causes a drop in the expression of all *PPARG* mRNAs, but with a more pronounced downregulation (about 80% reduction) of canonical transcripts (*p* = 0.0001). Accordingly, PPARGΔ5/cPPARG ratio shows a 2.6-fold increase in exposed cells (Figure 2A, *p* = 0.0214; lower left panel). In mature adipocytes—differentiated in vitro from hMSCs (T = 21d)—exposed to LPS-activated MΦ (THP-1)-conditioned media, a significant drop in the levels of cPPARG (about 95% reduction; *p* = 0.0038) and PPARGΔ5 (about 91% reduction; *p* = 0.0004) transcripts is observed (Figure 2A, lower right panel). However, likewise mature murine adipose cells (Figure 1C), PPARGΔ5/cPPARG ratio markedly increases (about 2-fold; *p* = 0.0115; Figure 2A, lower right panel). 

Considering that PPARGΔ5 inversely correlates with the adipogenic capacity of hMSCs [29] and that inflammation perturbs *PPARG* splicing in these cells (Figure 2A), we investigated which inflammatory stimuli may account for the unbalanced PPARGΔ5/cPPARG ratio. Hypertrophic AT is characterized by reduced adipogenesis and massive increase of pro-inflammatory macrophages by both infiltration and local proliferation [34]. Thus, we exposed hMSCs to the conditioned media of ex vivo-isolated human primary monocytes polarized toward two opposite distinct fates: pro-inflammatory (i.e., LPS/IFNγ-induced MΦ) or anti-inflammatory (IL10-induced MΦ) macrophages. As expected by our previous observation (Figure 2A), hMSCs exposed to the secretome of human primary pro-inflammatory MΦ display a strong transcriptional repression of the entire *PPARG* locus (Figure 2B). Strikingly, a more pronounced reduction is observed for canonical *PPARG* transcripts (about 60% reduction; *p* = 0.0001; Figure 2B, left panel), resulting in a 1.5-fold increase of PPARGΔ5/cPPARG ratio (*p* = 0.0208; Figure 2B, right panel). On the other hand, the secretome of primary anti-inflammatory MΦ—known to promote adipocyte differentiation [35]—induces a 2-fold increase of both canonical and dominant negative *PPARG* transcripts (*p* = 0.0061 and *p* = 0.076 respectively; Figure 2B, left panel), with no effects on their relative ratio (*p* = 0.9228; Figure 2B, right panel).

To exclude that any variation observed in *PPARG* transcripts’ abundance is due to a different half-life, a mRNA stability assay was performed by culturing hMSCs in the presence of growing amounts of the transcription inhibitor actinomycin D (ActD). Even at higher ActD doses, a similar degradation kinetic is observed for all *PPARG* transcripts (Figure 2C, left panel), without any effect on their relative ratio (Figure 2C, right panel), thus excluding differential mRNA decay as a possible source of bias.

Overall, our in vitro settings reveal that a pro-inflammatory microenvironment affects both *PPARG* expression and splicing, providing new insights into the mechanisms underlying the link between impaired adipogenesis and inflammation in AT.

### 3.3. TNFα Alters PPARG Splicing in Human Mesenchymal Stem Cells

**Figure 3:**
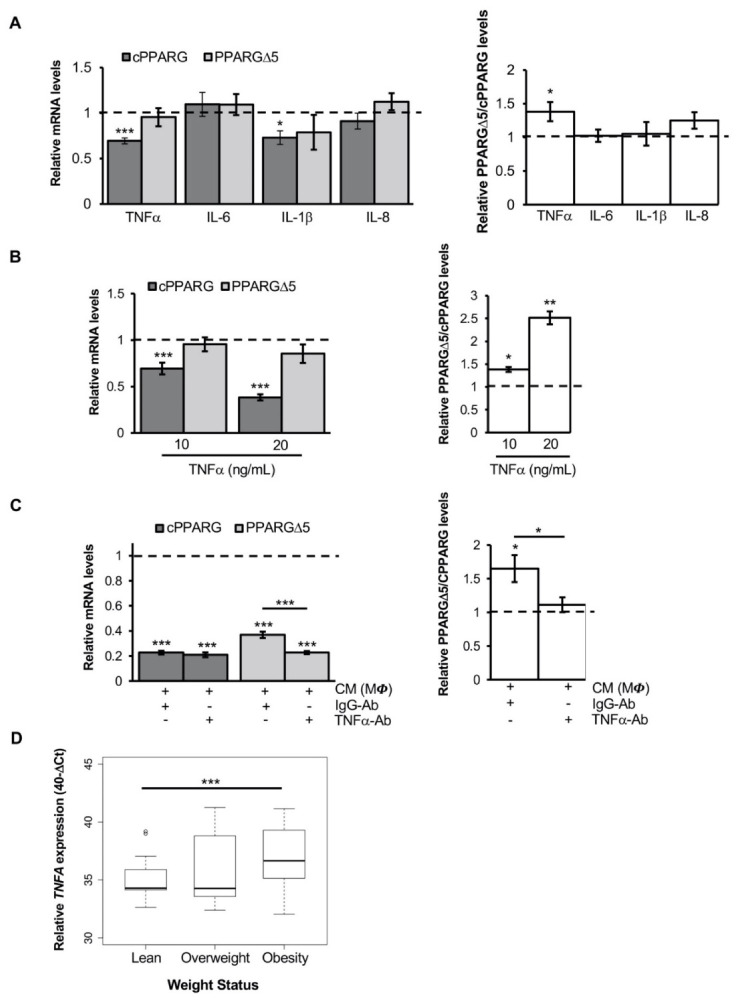
TNFα increases the PPARGΔ5/cPPARG ratio in adipocyte precursor cells and correlates with PPARGΔ5 expression in obese patients: (**A**) Relative mRNA quantification (qPCR) of cPPARG, PPARGΔ5 (left panel) and the PPARGΔ5/cPPARG ratio (right panel) in undifferentiated hMSCs treated for 24 h with 10 ng/mL of human recombinant TNFα, IL-6, IL-1β or IL-8 cytokines. hMSCs treated with vehicle (i.e., PBS) were used as reference samples (dotted lines) and *PPIA* as reference gene. Data are reported as mean ± SEM of at least three independent experiments. * *p* ≤ 0.051 and *** *p* ≤ 0.001. (**B**) Relative mRNA quantification (qPCR) of cPPARG, PPARGΔ5 (left panel) and the PPARGΔ5/cPPARG ratio (right panel) in hMSCs at undifferentiated stage treated for 24 h with 10 or 20 ng/mL of human recombinant TNFα cytokine. hMSCs treated with vehicle (i.e., PBS) were used as reference samples (dotted lines) and *PPIA* as reference gene. Data are reported as mean ± SEM of at least three independent experiments. * *p* ≤ 0.05, ** *p* ≤ 0.01 and *** *p* ≤ 0.001. (**C**) Relative mRNA quantification (qPCR) of cPPARG, PPARGΔ5 (left panel) and the PPARGΔ5/cPPARG ratio (right panel) in undifferentiated hMSCs treated for 24 h with conditioned medium (CM) of LPS-activated MΦ (THP-1) plus 0.5 mg/mL of human neutralizing antibody against TNFα or anti-IgG1 Isotype. hMSCs treated with control medium supplemented with antibody against anti-IgG1 Isotype were used as reference samples (dotted lines). *PPIA* was used as the reference gene. Data are reported as mean ± SEM of at least three independent experiments. * *p* ≤ 0.05 and *** *p* ≤ 0.001. (**D**) Boxplot showing *TNFA* levels in three different subgroups of individuals from the German cohort, classified according to their BMI in lean (n = 14), overweight (n = 17) and obesity (n = 24). Data are reported as 40-ΔCt value ± DEVST. *RPS23* was used as reference gene. *** *p* ≤ 0.001.

To identify which soluble factors released by pro-inflammatory macrophages are able to affect *PPARG* splicing, primary human monocytes were differentiated into macrophages and then polarized into pro-inflammatory and anti-inflammatory by LPS/IFNγ or IL-10 treatments, respectively. In line with previous studies [13,16,33,36], as shown in Figure S2A, we assessed higher levels of IL-1β, IL-6, IL-8 and TNFα in the conditioned media of LPS/IFNγ-induced MΦ (both vs. not polarized and IL10-induced MΦ),. Thus, *PPARG* expression and splicing pattern were analyzed in human mesenchymal precursors treated with all the above-mentioned cytokines. HMSCs exposed to human recombinant IL-6 or IL-8 do not display any variation in the expression—nor in the splicing pattern—of *PPARG* (Figure 3A, left panel). Differently, cPPARG mRNA is significantly reduced upon treatment with TNFα or IL-1β (about 40%, *p* = 0.001, and 30%, *p* = 0.0221, reduction respectively; Figure 3A, left panel). Thus, it is noteworthy that only TNFα significantly affects *PPARG* splicing, increasing the PPARGΔ5/cPPARG ratio (1.4-fold, *p* = 0.0272; Figure 3A, right panel). Similar results have been obtained by treating 3T3-L1 cells with murine recombinant TNFα and IL-1β (Figure S2B). Moreover, an high amount of TNF*α* was observed only in the conditioned media of mouse and human in vitro cultured macrophages (Figure S2C,D), able to increase PPARGΔ5/cPPARG *ratio* in adipocyte precursor cells and mature adipocytes (Figure 1C and Figure 2A). Interestingly, a suppressive dose-dependent effect is observed on cPPARG expression—but not on PPARGΔ5—when hMSCs are exposed to increasing TNFα doses (Figure 3B, left panel). Whereas the exposure to the highest TNFα amount (50ng/mL) causes massive cell death (data not shown), TNFα sublethal dose (20ng/mL) markedly increases the PPARGΔ5/cPPARG ratio (2.5-fold, *p* = 0.0123; Figure 3B, right panel). Notably, the addition of recombinant neutralizing anti-TNFα antibody (IgG) to the conditioned media of LPS-activated MΦ (THP-1) does not restore the expression of cPPARG, but reduces PPARGΔ5 levels (Figure 3C, left panel) reverting the TNFα-induced alteration on *PPARG* splicing in hMSCs (Figure 3C, right panel). 

Moreover, to investigate ex vivo the correlation between TNFα and *PPARG* splicing alteration, we evaluated *TNFA* expression in the subcutaneous adipose tissue (SAT) biopsies of a subset of lean, overweight and obese individuals (n = 56) from a German cohort, described in our previous studies [29,30]. As expected, *TNFA* mRNA expression was markedly higher in the obese group (vs. lean individuals; *p* = 0.0018; Figure 3D), in line with the increasing accumulation of pro-inflammatory macrophages in AT. Of note, we observed a mild positive correlation between *TNFA* expression and PPARGΔ5 mRNA levels (r = 0.45, *p* = 0.027; Figure S3A) only in the SAT of obese individuals, whereas no correlation was observed between *TNFA* and *PPARG* canonical transcripts (r = 0.19, *p* = 0.37; Figure S3B). These data reveal a previously unrecognized effect of TNFα on *PPARG* splicing, suggesting it as a relevant pro-inflammatory molecule able to negatively affect the transcription and splicing at the *PPARG* locus. Thus, although the transcriptional repression induced by TNFα on the *PPARG* gene has already been reported [21,22], our data reveal that TNFα does not equally affect all *PPARG* transcripts, determining an altered balance of canonical and dominant negative isoforms.

### 3.4. TNFα Modifies PPARG Splicing through PI3K/Akt Signaling

**Figure 4:**
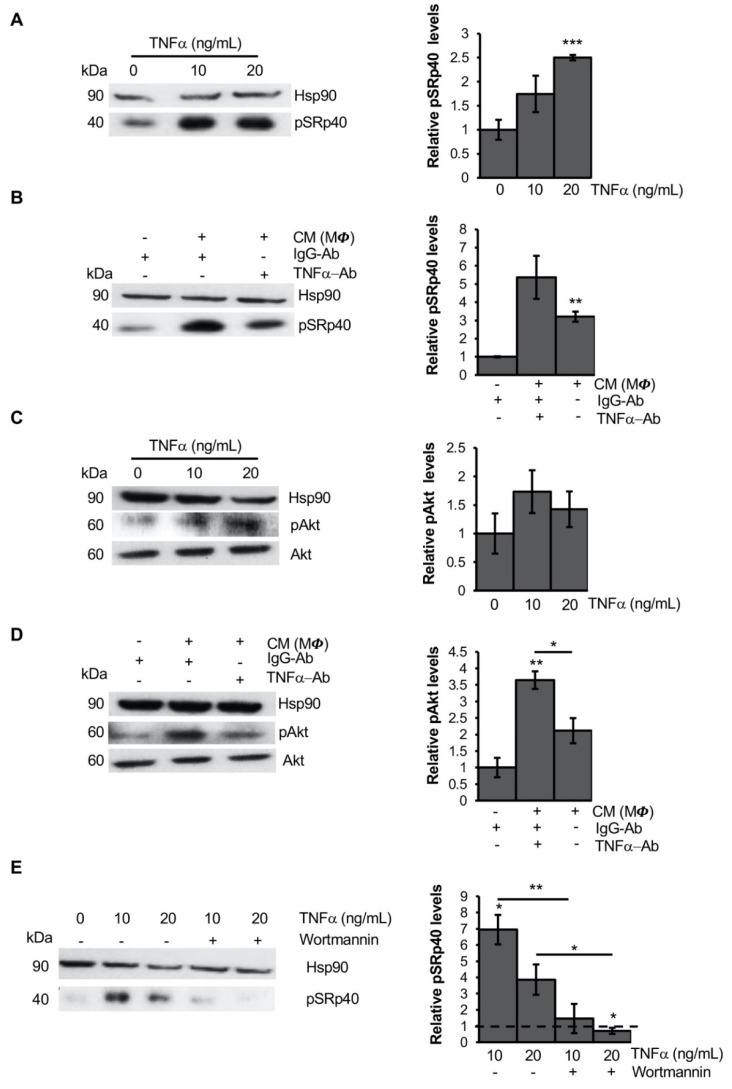
TNFα increases SRp40 phosphorylation through AKT: (**A**) Representative autoradiograph of Western blot analysis (left panel) of SRp40 phosphorylation (i.e., pSRp40) levels in hMSCSc at T = 0 h treated for 30 min with 10 or 20 ng/mL of TNFα cytokine or with vehicle (i.e., PBS). Hsp90 was used as a loading control. Bar graphs (right panel) report relative pSRp40 levels normalized on Hsp90 expression (pixel density analysis of Western blots). Data are representative of the mean ± SEM of three independent experiments. *** *p* ≤ 0.001. (**B**) Representative Western blot of pSRp40 (left panel) on lysates from undifferentiated hMSCs treated for 24 h with conditioned medium (CM) of LPS-activated MΦ (THP-1) or control medium plus 0.5 mg/mL of human neutralizing antibody against TNFα or anti-IgG1 Isotype. Hsp90 was used as loading control. Bar graphs (right panel) report relative pSRp40 levels normalized on Hsp90 expression (pixel density analysis of Western blots). Data are representative of the mean ±SEM of three independent experiments. ** *p* ≤ 0.01. (**C**) Representative immunoblotting of Akt phosphorylation (i.e., pAkt) and total Akt levels (right panel) in hMSCSc at T = 0 h treated for 30 min with 10 or 20 ng/mL of TNFα cytokine or with vehicle (i.e., PBS). Hsp90 was used as loading control. Bar graphs (right panel) report relative pAkt levels normalized on Hsp90 and total Akt expression (pixel density analysis of Western blots). Data are representative of the mean ± SEM of three independent experiments. (**D**) Representative autoradiograph of Western blot analysis (left panel) of pAkt and total Akt levels in undifferentiated hMScs treated for 24 h with the CM of LPS-activated MΦ (THP-1) or control medium plus 0.5 mg/mL of human neutralizing antibody against TNFα or anti-IgG1 Isotype. Hsp90 was used as loading control. Bar graphs (right panel) report relative pAkt levels normalized on Hsp90 and total Akt expression (pixel density analysis of Western blots). Data are representative of the mean ± SEM of at least three independent experiments. * *p* ≤ 0.05 and ** *p* ≤ 0.01. (**E**) Representative immunoblotting of pSRp40 (left panel) in undifferentiated hMSCs treated with Wortmannin or vehicle (i.e., DMSO) in combination and not with 10 or 20 ng/mL of TNFα cytokine. Hsp90 was used as loading control. Bar graphs (right panel) report relative pSRp40 levels normalized on Hsp90 expression (pixel density analysis of Western blots). Data are representative of the mean ± SEM of at least three independent experiments. * *p* ≤ 0.05 and ** *p* ≤ 0.01.

Since TNFα does not equally repress all *PPARG* transcripts—affecting only cPPARG levels—(Figure 3A,B) and canonical and alternative *PPARG* mRNAs have similar stability (Figure 2C), we hypothesized a direct effect of TNFα on members of the splicing machinery. In line with this hypothesis, TNFα is known to induce the splicing of different genes by phosphorylating multiple SR proteins [37,38,39]. Moreover, we previously reported the contribution of ASF2/SF2 to *PPARG* exon 5 skipping [29]. Therefore, we evaluated SR proteins phosphorylation in hMSCs exposed to TNFα, observing a pronounced phosphorylation of SRp40 (alias *SRSF5;* Figure 4A). Otherwise, we did not detect marked ASF/SF2 phosphorylation levels. Moreover, a dose-dependent increase of pSRp40 was observed (Figure 4A). Similarly, the conditioned media collected from LPS-activated MΦ (THP-1) are able to induce SRp40 phosphorylation (Figure 4B), which is reduced—but not completely abolished—by the addition of neutralizing anti-TNFα IgG (Figure 4B). This finding suggests that other MΦ-secreted pro-inflammatory factors may sustain SRp40 phosphorylation in the AT. 

Furthermore, we attempted to verify how TNFα promotes SRp40 phosphorylation in hMSCs. Interestingly, PI3K/Akt signaling—a known target of TNFα [40,41]—has been reported to induce alternative splicing of *PKCbII* gene via SRp40 phosphorylation [42]. Hence, we hypothesized that the stimulation of the PI3K/Akt pathway may mediate the effects of TNFα on SRp40. According to this hypothesis, pAkt levels (Ser473) significantly increase upon TNFα exposure (Figure 4C). Moreover, the addition of neutralizing TNFα antibody to the conditioned media of LPS-activated MΦ (THP-1) is able to reduce Akt phosphorylation (Figure 4D). Accordingly, PI3K inhibition – induced by cell treatment with wortmannin—blocks Akt phosphorylation and in turn TNFα-mediated phosphorylation of SRp40 (Figure 4E and Figure S4A).

**Figure 5:**
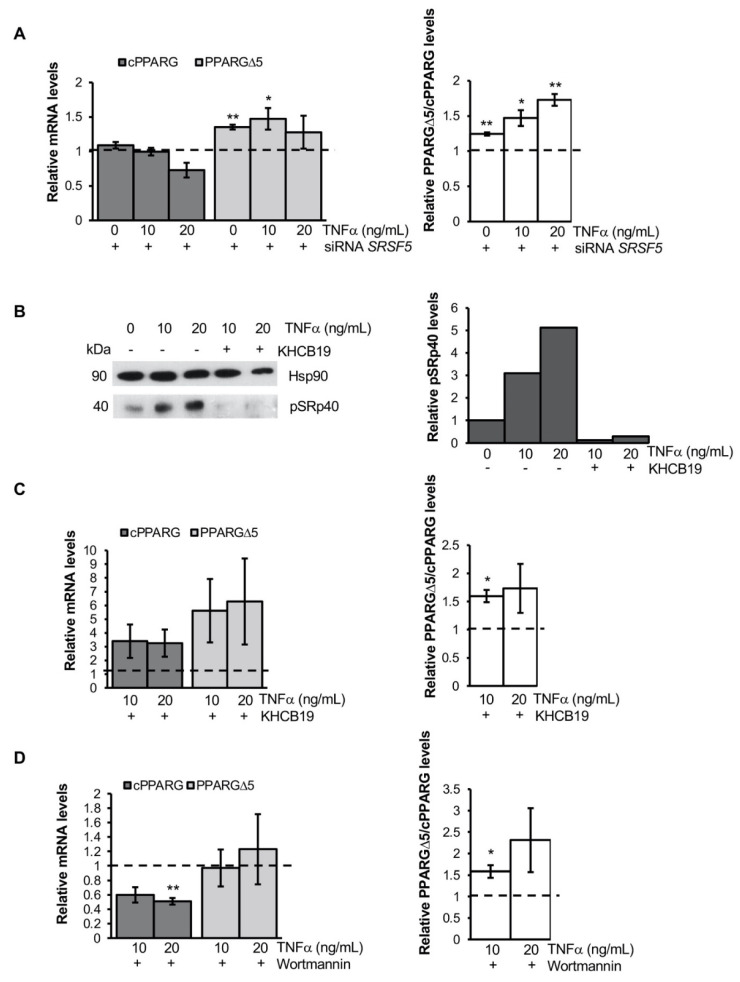
SRp40 is involved in the *PPARG* splicing modulation induced by TNFα: (**A**) Relative mRNA quantification (qPCR) of cPPARG, PPARGΔ5 (left panel) and the PPARGΔ5/cPPARG *ratio* (right panel) in hMSCs at the undifferentiated stage knock out for *SRSF5* and treated for 24 h with 0, 10 or 20 ng/mL of human recombinant TNFα cytokine. hMSCs at 0 h transfected scrambled and treated for 24 h with 0, 10 or 20 ng/mL of human recombinant TNFα cytokine were used as reference samples (dotted lines). *PPIA* was used as the reference gene. Data are reported as mean ± SEM of at least three independent experiments. * *p* ≤ 0.05 and ** *p* ≤ 0.01. (**B**) Western blot of SRp40 phosphorylation (i.e., pSRp40; left panel) levels in hMSCs at T = 0 h treated with wortmannin or vehicle (i.e., DMSO) in combination and not with 10 or 20 ng/mL of TNFα cytokine. Hsp90 was used as loading control. Bar graphs (right panel) report relative pSRp40 levels normalized on Hsp90 expression (pixel density analysis of Western blot). (**C**) Relative mRNA quantification (qPCR) of cPPARG, PPARGΔ5 (left panel) and the PPARGΔ5/cPPARG *ratio* (right panel) in hMSCs at the undifferentiated stage treated with 10 or 20 ng/mL of human recombinant TNFα cytokine in combination or not with KHCB19 inhibitor. hMSCs at 0h treated for 24 h with 10 or 20 ng/mL of human recombinant TNFα cytokine and with vehicle (i.e., DMSO) were used as reference samples (dotted lines). *PPIA* was used as the reference gene. Data are reported as mean ±SEM of at least three independent experiments. * *p* ≤ 0.05. (**D**) Relative mRNA quantification (qPCR) of cPPARG, PPARGΔ5 (left panel) and the PPARGΔ5/cPPARG *ratio* (right panel) in hMSCs at the undifferentiated stage treated with 10 or 20 ng/mL of human recombinant TNFα cytokine and with or without Wortmannin inhibitor. hMSCs at 0 h treated for 24 h with 10 or 20 ng/mL of human recombinant TNFα cytokine and with vehicle (i.e., DMSO) were used as reference samples (dotted lines). *PPIA* was used as the reference gene. Data are reported as mean ± SEM of at least three independent experiments. * *p* ≤ 0.05 and ** *p* ≤ 0.01.

In light of these data, we first evaluated the impact of SRp40 silencing on TNFα-mediated *PPARG* splicing in hMSCs. Surprisingly, *SRSF5* knockdown (Figure 5A and Figure S4B,C), in cells treated with TNFα or the conditioned media of LPS-activated MΦ (THP-1), significantly increases *PPARG* exon 5 skipping and, in turn, the PPARGΔ5/cPPARG *ratio*. This unexpected result suggests that SRp40 is required to balance the inclusion/exclusion rate of *PPARG* exon 5 in the presence of inflammatory *stimuli.*

Moreover, it is documented that different SR proteins may cooperatively work to regulate the alternative splicing [43,44,45]. Hence, to explore whether other SR proteins may account for *PPARG* exon 5 skipping, we simultaneously blocked the activity of multiple SR proteins, including SRp40. TNFα-treated hMSCs were exposed to drugs (i.e., KHCB19 and SRPIN340) inhibiting two distinct classes of protein kinases—i.e., Cyclin-dependent kinases 1–4 (Clk) and Serine/arginine rich protein kinases 1–2 (Srpk)—which phosphorylate (and activate) SR proteins. Moreover, using wortmannin, we blocked PI3K/Akt signaling, responsible for Clk- and Srpk-mediated SR phosphorylation [44]. Interestingly, the inhibition of Clk by KHCB19 in TNFα-treated hMSCs, induces a strong increase of PPARGΔ5 levels and of PPARGΔ5/cPPARG *ratio* (Figure 5B,C), likewise the inhibition of Srpk1 and Srpk2 proteins by SRPIN340 (Figure S4D). Similarly, PI3K inhibition by wortmannin in presence of TNFα was sufficient to increase the PPARGΔ5/cPPARG *ratio* (Figure 5D).

Thus, these results further support the role of PI3K/Akt signaling in linking inflammation to *PPARG* splicing and suggest that the inhibition of SR proteins facilitates the activity of other competing splicing factors—such as heterogeneous nuclear ribonucleoproteins (hnRNPs) [46,47,48]—in the modulation of *PPARG* exon 5 inclusion/exclusion.

## 4. Discussion

Insulin resistance in individuals with hypertrophic obesity is causally associated with low-grade chronic inflammation, characterized by the infiltration of T cells, macrophages and other immune cells [4,5,6,7,8,9,10]. The release of cytokines and chemokines by pro-inflammatory and metabolically activated macrophages [12,14,15] has a negative impact on the expression and activity of PPARγ [19,20,21,22]. This nuclear receptor has a well-recognized central role in differentiating new insulin-sensitive adipocytes, in keeping the metabolic homeostasis of adipose tissue, in repressing inflammatory genes (e.g., *TNF*α, *iNOS*, *MMP9*) [23,24,25,26,27,28,49] and in dictating monocyte polarization toward an anti-inflammatory phenotype [50,51,52]. Hence, the repressing effect induced by the inflammation on PPARγ generates a self-sustained vicious cycle, contributing to the impairment of neo-adipogenesis, which in turn promotes adipocyte hypertrophy, as well as local and global inflammation [12,18]. We previously described a marked overexpression of the dominant-negative isoform of PPARγ, PPARγΔ5, in the AT of obese/diabetic patients and its ability to impair adipocyte differentiation and PPARγ activity in hypertrophic adipocytes [29,30]. Moreover, the increased PPARGΔ5/cPPARG *ratio* in SAT enriched of large adipocytes, as well as in our recently generated in vitro model of human hypertrophic-like adipocytes [30], further encouraged us to investigate the putative contribution of pro-inflammatory factors—highly secreted in hypertrophic AT—to the unbalance of *PPARG* isoforms. 

Herein, we aimed to dissect the impact of inflammatory *stimuli* on *PPARG* expression and splicing, considering that the relevance of the latter has been often underestimated. The identification, for the first time, that inflammatory factors, other than repressing *PPARG* expression can modulate—in vitro and in vivo—its splicing pattern, adds an interesting piece to the puzzling regulation of *PPARG*. In this regard, our results provide evidence of a marked increase of the PpargΔ5/cPparg *ratio*—paralleled by the repression of *Pparg*-regulated adipocyte markers—in the epididymal fat of LPS-treated mice, as well as in murine and human adipocytes exposed to macrophages-secreted pro-inflammatory factors. A similar effect on *PPARG* splicing is also observed in human mesenchymal stem cells, in which we recently described a role for PPARGΔ5 in reducing their adipogenic capacity [29]. Taken together, our data provide an intriguing insight in the molecular mechanisms through which the proinflammatory microenvironment contributes to the impaired neo-adipogenesis in hypertrophic AT, suggesting for *PPARG* splicing a role as a hub. The increased PPARGΔ5/cPPARG *ratio* in the SAT of overweight/obese individuals [29] and in lipid-engulfed adipocytes [30] further support this hypothesis. However, we cannot discern the relative contributions of functional isoforms, whose levels are reduced, and of increased dominant negative isoforms to the impairment of PPARγ activity leading to a diminished adipogenic differentiation.

Moreover, in line with the suggested role of TNFα as a key mediator of the effects induced by inflammation on preadipocytes and adipocyte differentiation [53,54], herein we reported TNFα—known to inhibit PPARγ activity through distinct mechanisms [21,22]—as a major pro-inflammatory cytokine able to alter *PPARG* splicing pattern. Ex vivo analyses on SAT biopsies revealed higher *TNFA* expression and a positive correlation with PPARGΔ5 levels only in obese (but not in lean) individuals, further supporting also in human hypertrophic adipocytes the link between inflammation and *PPARG* splicing alteration. Interestingly, the finding that canonical and alternative *PPARG* transcripts have the same half-life but respond differently to TNFα, as well as to the conditioned media of proinflammatory macrophages, supports a direct effect of inflammation on the splicing machinery. Of note, the alteration of spliceosome machinery due to altered levels of phosphorylated proteins involved in mRNA splicing has been recently reported even in the context of obesity and T2D [55,56,57]. Moreover, TNFα is known to dynamically modulate the inclusion/exclusion rate of alternative exons by enhancing the phosphorylation of multiple SR proteins [37,38,39]. In our experimental settings—i.e., in hMSCs exposed to MΦ-secreted pro-inflammatory factors—we found a TNFα-mediated increase of SRp40 phosphorylation, suggesting a still unexplored role for this splicing factor in *PPARG* exon 5 inclusion/exclusion. However, as we did not definitely establish the exact role of SRp40 in *PPARG* splicing, we cannot exclude that multiple SR proteins may cooperate in the modulation of *PPARG* exon 5 inclusion/exclusion balance and in turn to the generation of the truncated PPARγ isoform.

The finding that MΦ-secreted TNFα impairs PPARγ functionality by promoting the generation of dominant negative isoforms over canonical ones has relevance also from a therapeutic perspective. Indeed, the recently developed anti-inflammatory drugs, which specifically targeting pro-inflammatory MΦ, revealed to be very promising to ameliorate obesity-related inflammation and metabolic dysfunctions [58]. Moreover, the mechanisms underlying polarization of MΦ into pro-inflammatory and metabolically activated cells – rather than in anti-inflammatory MΦ—in the AT of obese patients [12,14,15] remain to be elucidated. Of note, human monocytes/macrophages express PPARγ dominant negative isoforms [29,59]. Hence, given the key role of PPARγ in monocyte polarization, we might speculate that autocrine (or paracrine) signaling in a hypertrophic AT microenvironment may affect *PPARG* transcripts abundance in AT-resident MΦ, contributing to disease etiology. 

In conclusion, our results demonstrate a novel mechanism through which inflammation impairs PPARγ activity in the AT. The imbalance among functional and dominant negative *PPARG* transcripts is likely to contribute to establishing healthy and unhealthy AT under positive energy intake. These data further indicate the relevance to target inflammation in adipose tissue of patients with hypertrophic obesity to minimize the detrimental effects that pro-inflammatory molecules have on PPARγ functionality in this tissue. 

## Supplementary Materials

The following are available online at https://www.mdpi.com/article/10.3390/cells11010042/s1, Figure S1: Conditioned media from mouse macrophages modulate the expression of cPparg and PpargΔ5 transcripts. Figure S2: Cytokines secretions by ex vivo and in vitro macrophages and their effects on *Pparg* splicing in 3T3-L1 cells. Figure S3: *TNFA* mRNA levels positive correlate with PPARGΔ5 levels in obese individuals. Figure S4: Inflammatory *stimuli* modulate *PPARG* splicing through SRp40 phosphorylation. Table S1: List of primers sequence of gene analyzed by qPCR.
