# Supplementary material for: TNFα Mediates Inflammation-Induced Effects on PPARG Splicing in Adipose Tissue and Mesenchymal Precursor Cells
Source: Cells. 2021 Dec 24;11(1):42. doi: 10.3390/cells11010042 (PMC8750445; doi:10.3390/cells11010042)
Supplement: Supplementary file 1 [file cells-11-00042-s001.zip › cells-1356632-supplementary.pdf]

Supplementary Material and Methods

The experimental protocols used for activation and differentiation of J774.A1 and THP-1 and described in “Material and Methods” section, were set starting from similar protocols employed by Starr et al., 2018, Huang et al., 2012, Schutte et al., 2009 (for THP-1) and Wang et al., 2019, Nativel et al., 2017, Kheder et al., 2016, Bartosh et al., 2014, Huang et al., 2012 (for J774.A1), overall assessing the secretion of pro-inflammatory cytokines and/or surface markers of obtained - activated and differentiated - cells.
Similarly, the adipocyte differentiation of 3T3-L1 cell lines in presence of J774.A1 conditioned medium - described in “Material and Methods” section - was experimentally defined in line with protocols described by Constant et al., 2006.

Supplementary References

Bartosh TJ, Ylostalo JH. Macrophage Inflammatory Assay. Bio Protoc. 2014 Jul 20;4(14):e1180. doi: 10.21769/bioprotoc.1180. PMID: 27570796; PMCID: PMC4999258.

Constant VA, Gagnon A, Landry A, Sorisky A. Macrophage-conditioned medium inhibits the differentiation of 3T3-L1 and human abdominal preadipocytes. Diabetologia. 2006 Jun;49(6):1402-11. doi: 10.1007/s00125-006-0253-0. Epub 2006 Apr 12. PMID: 16609875.

Daigneault M, Preston JA, Marriott HM, Whyte MK, Dockrell DH. The identification of markers of macrophage differentiation in PMA-stimulated THP-1 cells and monocyte-derived macrophages. PLoS One. 2010 Jan 13;5(1):e8668. doi: 10.1371/journal.pone.0008668. PMID: 20084270; PMCID: PMC2800192.

Huang H, Fletcher A, Niu Y, Wang TT, Yu L. Characterization of lipopolysaccharide-stimulated cytokine expression in macrophages and monocytes. Inflamm Res. 2012 Dec;61(12):1329-38. doi: 10.1007/s00011-012-0533-8. Epub 2012 Jul 28. PMID: 22842767.

Kheder RK, Hobkirk J, Stover CM. In vitro Modulation of the LPS-Induced Proinflammatory Profile of Hepatocytes and Macrophages- Approaches for Intervention in Obesity? Front Cell Dev Biol. 2016 Jun 22;4:61. doi: 10.3389/fcell.2016.00061. PMID: 27446914; PMCID: PMC4916220.

Nativel B, Couret D, Giraud P, Meilhac O, d'Hellencourt CL, Viranaïcken W, Da Silva CR. Porphyromonas gingivalis lipopolysaccharides act exclusively through TLR4 with a resilience between mouse and human. Sci Rep. 2017 Nov 17;7(1):15789. doi: 10.1038/s41598-017-16190-y. PMID: 29150625; PMCID: PMC5693985.

Schutte RJ, Parisi-Amon A, Reichert WM. Cytokine profiling using monocytes/macrophages cultured on common biomaterials with a range of surface chemistries. J Biomed Mater Res A. 2009 Jan;88(1):128-39. doi: 10.1002/jbm.a.31863. PMID: 18260130; PMCID: PMC4070304.

Starr T, Bauler TJ, Malik-Kale P, Steele-Mortimer O. The phorbol 12-myristate-13-acetate differentiation protocol is critical to the interaction of THP-1 macrophages with Salmonella Typhimurium. PLoS One. 2018 Mar 14;13(3):e0193601. doi: 10.1371/journal.pone.0193601. PMID: 29538403; PMCID: PMC5851575.

Wang Z, Maruyama K, Sakisaka Y, Suzuki S, Tada H, Suto M, Saito M, Yamada S, Nemoto E. Cyclic Stretch Force Induces Periodontal Ligament Cells to Secrete Exosomes That Suppress IL-1β Production Through the Inhibition of the NF-κB Signaling Pathway in Macrophages. Front Immunol. 2019 Jun 20;10:1310. doi: 10.3389/fimmu.2019.01310. PMID: 31281309; PMCID: PMC6595474.

Figure S1

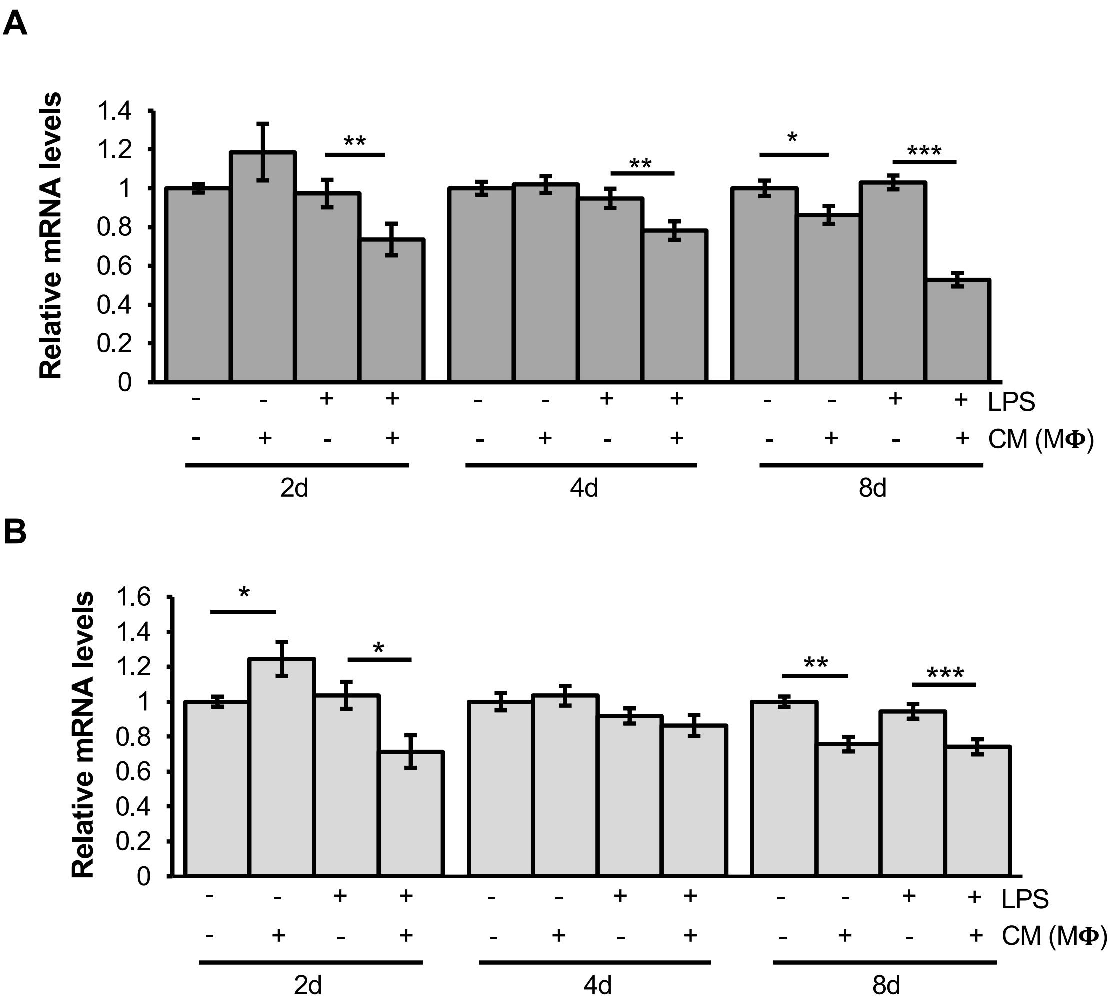
A
Relative mRNA levels
1.4
1.2
1
0.8
0.6
0.4
0.2
0
**
**
*
***
LPS
CM (MΦ)
2d
4d
8d
B
Relative mRNA levels
1.6
1.4
1.2
1
0.8
0.6
0.4
0.2
0
*
*
**
***
LPS
CM (MΦ)
2d
4d
8d

Figure S1: (A-B) Relative mRNA quantification (qPCR) of cPparg and PpargΔ5 (B) at different time points of 3T3-L1 adipocyte differentiation (i.e. 2, 4 and 8 days upon differentiation induction) carried out in presence of conditioned medium (CM) of J774.A1 macrophages (MΦ) activated and not with LPS. 3T3-L1 differentiated in mature adipocytes with control medium supplemented or not with LPS were used as reference samples. *36b4* was used as reference gene. Data are reported as mean ±SEM of at least six independent experiments. $^*p \leq 0.05$, $^{**}p \leq 0.01$, $^{***}p \leq 0.001$.

Figure S2

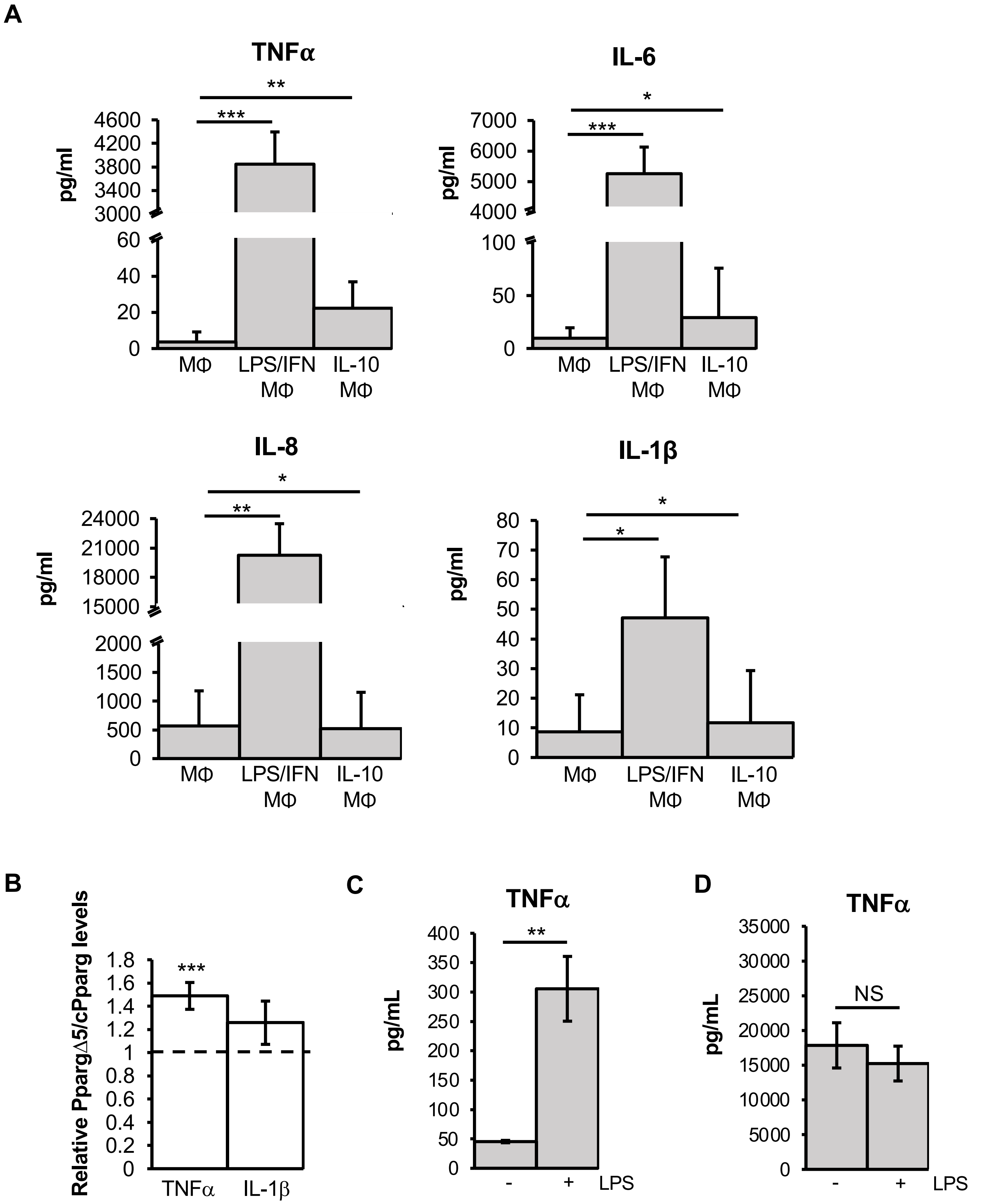
A
TNFα
IL-6
IL-8
IL-1β
pg/ml
MΦ
LPS/IFN MΦ
IL-10 MΦ
B
Relative PpargΔ5/cPparg levels
TNFα
IL-1β
C
TNFα
pg/mL
LPS
D
TNFα
pg/mL
NS
LPS

Figure S2: (A) Bar graphs reporting TNFα, IL-6, IL-1β or IL-8 cytokines levels secreted by monocyte-derived macrophages non-polarized (MΦ) or polarized in pro-inflammatory (i.e. LPS/IFN-induced MΦ) or anti-inflammatory (i.e. IL-10-induced MΦ) macrophages. The estimation of cytokines secreted amount (pg/mL) was performed by ELISA in the supernatant of macrophages from 3 different healthy donors. The differences in the intensity of the response and reported as ±SEM depend exclusively on the inter-individual variability. *p ≤ 0.05, **p ≤ 0.01, ***p ≤ 0.001. **(B)** Relative mRNA quantification (qPCR) of PpargΔ5/cPparg *ratio* in 3T3-L1 fibroblasts at undifferentiated stage treated for 24 hours with 10 ng/ml of mouse recombinant TNFα or IL-1β cytokines. 3T3-L1 cells at 0h treated with vehicle (i.e. PBS) were used as reference samples (dotted lines) and *36b4* as reference gene. Data are reported as mean ±SEM of at least three independent experiments. ***p ≤ 0.001. **(C-D)** Bar graphs reporting TNFα cytokine levels secreted by J774A.1 macrophages activated and not by LPS and by THP-1 differentiated by PMA in macrophages activated and not with LPS (D). The estimation of cytokines secreted amount (pg/mL) was performed by ELISA in the supernatant of macrophages. Data are reported as mean ±SEM of three independent experiments. **p ≤ 0.01, NS= not significant.

Figure S3

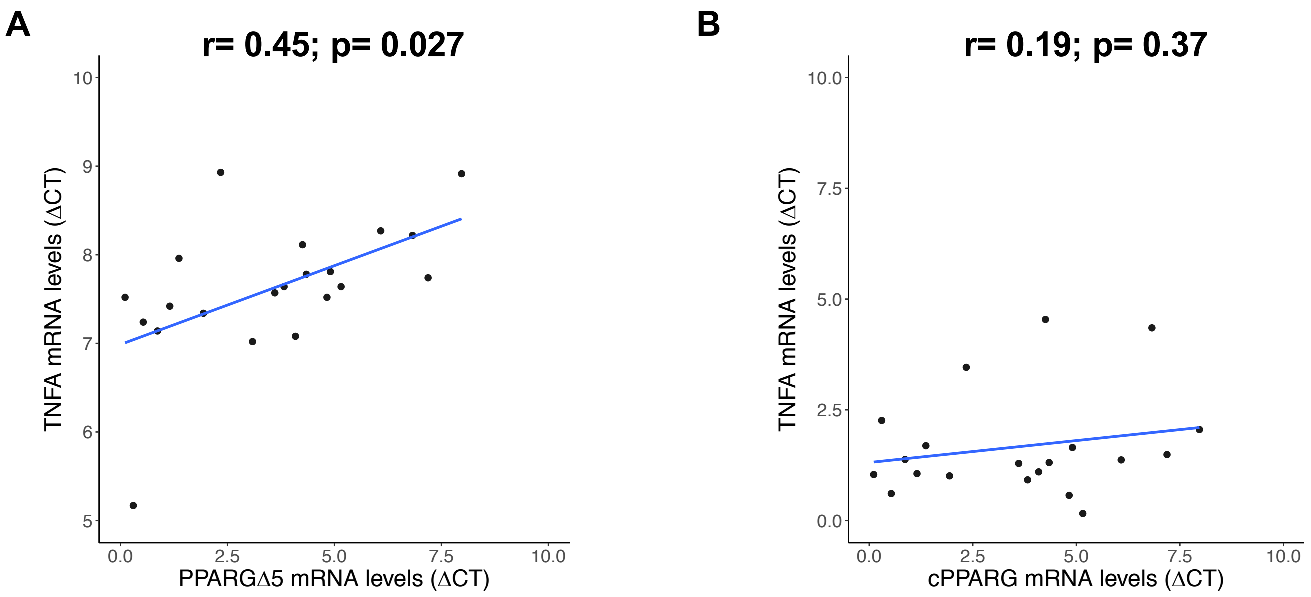
A
r= 0.45; p= 0.027
TNFA mRNA levels (ΔCT)
PPARGΔ5 mRNA levels (ΔCT)
B
r= 0.19; p= 0.37
TNFA mRNA levels (ΔCT)
cPPARG mRNA levels (ΔCT)

Figure S3: (A-B) Scatter plot reporting the correlation by linear regression analysis between *TNFA* mRNA levels and PPARGΔ5 (A) and cPPARG (B) expression in obese individuals from the German cohort (n=24). The average expression value of lean individuals was used as reference. *RPS23* was used as housekeeping gene. Pearson correlation coefficient (*r*) and p value (*p*) are shown.

Figure S4

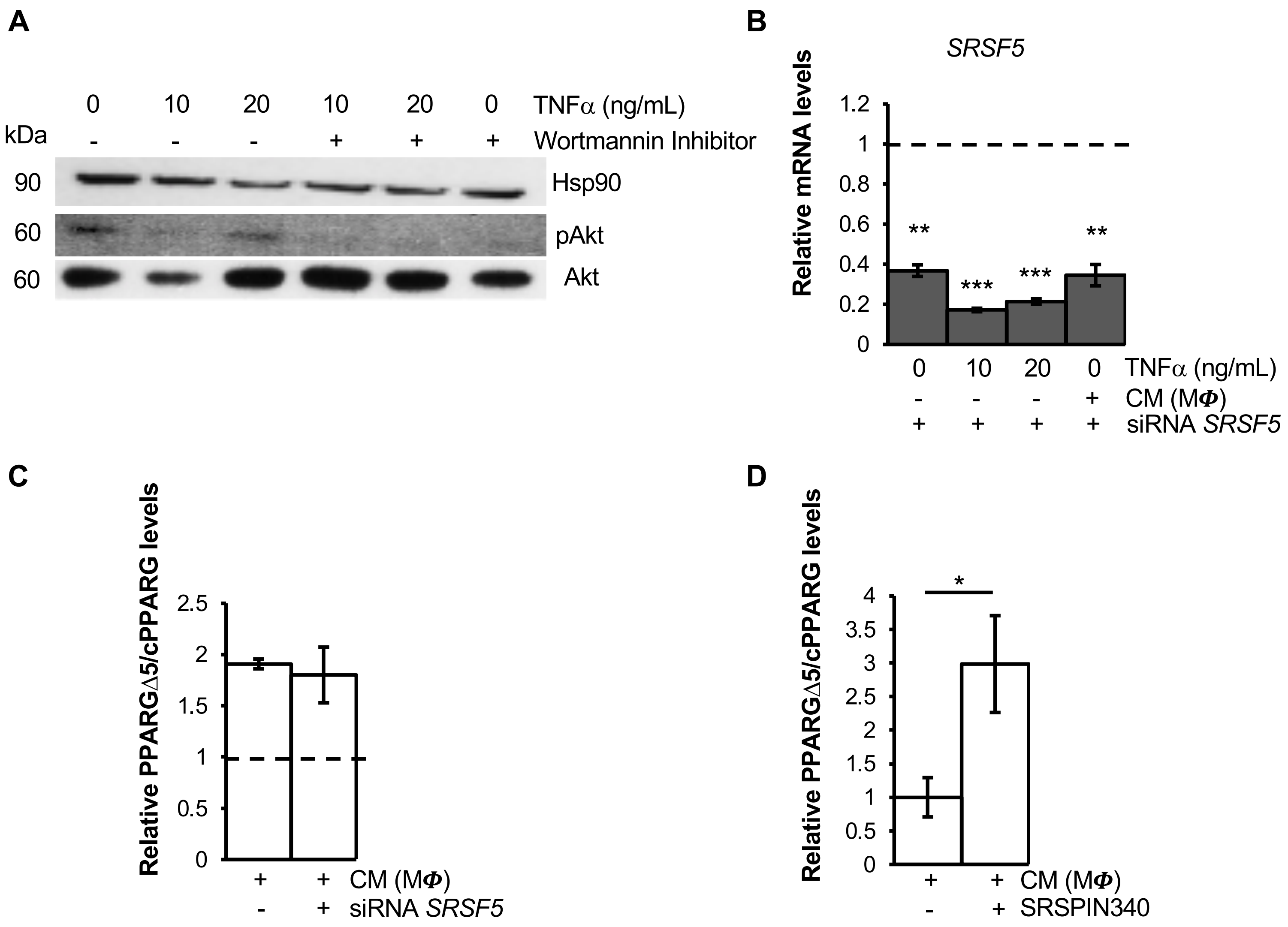

A
0
10
20
10
20
0
TNFα (ng/mL)
kDa
- - - + + +
Wortmannin Inhibitor
90
Hsp90
60
pAkt
60
Akt
B
SRSF5
Relative mRNA levels
1.2
1
0.8
0.6
0.4
0.2
0
**
***
***
**
0 10 20 0 TNFα (ng/mL)
- - - + CM (MΦ)
+ + + + siRNA SRSF5
C
Relative PPARGΔ5/cPPARG levels
2.5
2
1.5
1
0.5
0
+ + CM (MΦ)
- + siRNA SRSF5
D
Relative PPARGΔ5/cPPARG levels
*
4
3.5
3
2.5
2
1.5
1
0.5
0
+ + CM (MΦ)
- + SRSPIN340

Figure S4: (A) Representative immunoblotting of Akt phosphorylation (i.e. pAkt) and total Akt levels in undifferentiated hMSCs treated with Wortmannin or vehicle (i.e. DMSO) in combination and not with 10 or 20 ng/mL of TNFα cytokine. Hsp90 was used as loading control. (**B)** hMSCs were transiently silenced with small interfering RNA (siRNA) against *SRSF5* gene and treated for 24 hours with 0, 10 or 20 ng/ml of human recombinant TNFα cytokine or with conditioned medium (CM) of LPS-activated MΦ (THP-1). Bar graphs report the relative mRNA quantification (qPCR) of *SRSF5*. hMSCs at 0h transfected with scrambled and treated for 24 hours with 0, 10 or 20 ng/ml of human recombinant TNFα cytokine or with control medium were used as reference samples (dotted lines). *PPIA* was used as reference gene. Data are reported as mean ±SEM from at least three independent experiments. $^{**}p \leq 0.01$ and $^{***}p \leq 0.001$. (**C)** Relative mRNA quantification (qPCR) of PPARGΔ5/cPPARG *ratio* in hMSCs at undifferentiated stage silenced for *SRSF5* and treated for 24 hours with conditioned medium (CM) of LPS-activated MΦ (THP-1). hMSCs at 0h transfected with scrambled and treated for 24 hours with control medium were used as reference samples (dotted lines). *PPIA* was used as reference gene. (**D)** Relative mRNA quantification (qPCR) of PPARGΔ5/cPPARG *ratio* in hMSCs at undifferentiated stage treated for 24 hours with conditioned medium (CM) of LPS-activated MΦ (THP-1) plus SRPIN340 inhibitor. hMSCs at 0h treated for 24 hours with CM of MΦ plus vehicle (i.e. DMSO) were used as reference samples and *PPIA* as reference gene. Data are reported as mean ±SEM of at least three independent experiments. $^{*}p \leq 0.05$.

Table S1. List of primers sequence of gene analyzed by qPCR

Gene	Sense Primer (5′---3′)	Antisense Primer (5′---3′)	Melting Temperature
cPparg	ACAGGCCGAGAAGGAGAAG	TCTTGCACGGCTTCTACGG	60°
PpargΔ5	TGCCTTGCTGTGGGGATGT	AGCAAGCCTGGGCGGTTGA	60°
36b4	TCCAGGCTTTGGGCATCA	CTTTATCAGCTGCACATCACTCAGA	60°
Adipoq	AGAGAAGGGAGAGAAAGGAG	GCCAGTGCTGCCGTCATAAT	60°
Slc2a4	ACGAGCTGGACGACGGACA	AGCTCTGCCACAATGAACCA	60°
Cd36	GTCCTATTGGCCAAGCTATT	GCAAATGTCAGAGGA AAAGAA	60°
cPPARG	GAGAAGGAGAAGCTGTTGGC	ATGGCCACCTCTTTGCTCT	60°
PPARGΔ5	CTTGCAGTGGGGATGTCTCA	CAGCAAACCTGGGCGGTTGA	60°
PPIA	TACGGGTCCTGGCATCTTGT	GGTGATCTTCTTGCTGGTCT	60°
TNFA	CGAACCCCGAGTGACAAGC	GCTGATGGTGTGGGTGAGG	60°
